# Hyperthermia Targeting the Tumor Microenvironment Facilitates Immune Checkpoint Inhibitors

**DOI:** 10.3389/fimmu.2020.595207

**Published:** 2020-11-09

**Authors:** Zihui Li, Jie Deng, Jianhai Sun, Yanling Ma

**Affiliations:** Oncology Department, The Third People’s Hospital of Hubei Province, Affiliated Hospital of Jianghan University, Wuhan, China

**Keywords:** hyperthermia, tumor microenvironment, immune checkpoint inhibitors, combined therapy, synergetic effect

## Abstract

Immune checkpoint inhibitors (ICIs) have ushered in a new era of cancer therapy; however, ICIs are only effective in selective patients. The efficacy of ICIs is closely related to the tumor microenvironment. Fever for a long time was thought to directly regulate the immune response, and artificial “fever” from hyperthermia modulates the tumor immune microenvironment by providing danger signals with heat shock proteins (HSPs) as well as subsequent activation of immune systems. Encouraging results have been achieved in preclinical studies focused on potential synergetic effects by combining hyperthermia with ICIs. In this review, we summarized a cluster of immune-related factors that not only make hyperthermia a treatment capable of defending against cancer but also make hyperthermia a reliable treatment that creates a type I-like tumor microenvironment (overexpression of PD-L1 and enrichment of tumor infiltrating lymphocytes) in complementary for the enhancement of the ICIs. Then we reviewed recent preclinical data of the combination regimens involving hyperthermia and ICIs that demonstrated the combined efficacy and illustrated possible approaches to further boost the effectiveness of this combination.

## Introduction

Immune checkpoint inhibitors (ICIs) aim to reverse the immunosuppressive tumor microenvironment (TME) have ushered in a new era of cancer treatment. Efficacy of ICIS-based cancer immunotherapy relies on the immune status in TME. TME is composed of tumor cells, immune/inflammatory cells, stromal cells, blood/lymph vessels, cytokines, secreted proteins, RNAs, and small organelles (1). Through signal transduction and intercellular interactions, TME constitutes and modulates the cancer-immunity cycle (2). Based on the immune status of TME, tumors can be classified as “cold” and “hot” in which “cold” tumors often have a low response rate to anti-PD-1/PD-L1 mAb due to reduced tumor mutation, less T-cell infiltration, less PD-L1 expression and enrichment in immunosuppressive cells (3). Anti-angiogenesis treatment, radiotherapy, or chemotherapy increases the treatment efficacy of ICIs by transforming the immune status of TME through exposure of tumor-specific antigens, normalization of the endothelium, attraction of immune cells, *etc*. (4–6) Hyperthermia can also modulate the immune status of TME and influence the immune system through cytotoxic effects of high temperatures (7).

Hyperthermia is a method of killing cancer cells or impeding their growth by increasing tissue temperature with an external heat source for a certain period of time. Hippocrates, the father of modern medicine said, “Those who cannot be cured by surgery can be cured by heat. Those who cannot be cured by heat, they are indeed incurable.” Early studies found that fever was correlated with spontaneous tumor regression. Since the last century, hyperthermia has been widely used for patients with both locoregional and advanced cancers in prostate cancer, melanoma, bladder cancer, esophageal cancer, and cervical cancer. The combination of hyperthermia and radio-/chemotherapy has also shown effectiveness for tumor control in numerous clinical studies. Hyperthermia can be classified as local (microwaves, radio waves, or ultrasound), regional (hyperthermic intraperitoneal chemotherapy), and whole body hyperthermia depending on the organ to be targeted. While based on temperature ranges, hyperthermia can be classified as fever-range temperature hyperthermia (39–40°C), mild temperature hyperthermia (heat shock temperature, 41–43°C), and thermal ablation (cytotoxic temperature, >43°C). Magnetic nanoparticle hyperthermia, cryo-thermal therapy, and photothermal therapy are newly developed treatments that also belong to the category of hyperthermia.

Hyperthermia modulates the immune status of tumor microenvironment by providing danger signals with HSPs as well as subsequent activation of immune systems. The immunomodulatory effects not only make hyperthermia a treatment capable of defending against cancer but also make hyperthermia a reliable treatment that creates a type I-like tumor microenvironment (overexpression of PD-L1 and enrichment of tumor infiltrating lymphocytes) in complementary for the enhancement of the ICIs. Below, we summarize a cluster of immune-related factors that are inducible by hyperthermia, highlighting the complementary effect of hyperthermia on immunogenicity and immunoreactivity in the tumor microenvironment for the enhancement of ICIs. Then we reviewed recent preclinical data of the combination regimens involving hyperthermia and ICIs that demonstrated the combined efficacy and illustrated possible approaches to further boost the effectiveness of this combination.

## Hyperthermia Increases Tumor DNA Damage

The T cell-based immune system frequently responds to neoantigens that arise as a consequence of accumulated DNA damage, known as the tumor mutation burden (8, 9). High tumor mutation achieves a higher response rate to PD-1/PD-L1 mAb and gets a higher objective tumor remission rate. Hyperthermia directly and indirectly induces DNA damage in addition to interacting and interfering with various DNA repair cascades, all of which contribute to mutations in the tumor genome and the production of neoantigens (8, 10). Briefly, hyperthermia can directly induce the DNA damage response by promoting single stranded break (SBS), double stranded break (DBS), histone H2AX with phosphorylated C-terminal serines (*γ*-H2AX) foci formation and ataxia-telangiectasia mutated protein(ATM) phosphorylation and decelerating DNA replication and repair (downregulate DNA polymerases and topoisomerases activity), and indirectly activate DNA damage response and induce tumor suppressor alternative reading frame (ARF) by promoting ROS production, cell cycle arrest, cell cycle checkpoint arrest, cell death, decelerate DNA replication. Moreover, hyperthermia can significantly promote DNA damage in tumor stem cells that are resistant to most classical treatment regimens, which would be more effective for the formation of tumor neoantigens (11). In addition, exosomes extracted from heat-stressed tumor cells (HS-TEX) induce a bystander effect that can transform DNA damage from heat-stressed tumor cells to the non-heated ones (12). The enhanced irreversible cellular DNA damage accumulation was further proven by that hyperthermia is applied as a complement in treatment combining chemotherapy or irradiation to induce irreversible cellular DNA damage (13). Nevertheless, the identification of neoantigens requires the mapping of tumor-specific genetic aberrations using whole-exosome sequencing, *in silico* predictions, mass spectrometry, and T cell assays (9).

## Hyperthermia Is a Strong ICD Inducer

Despite mutations and neoantigens for the potential initiation of immunity, only immunogenic characteristic defined by immunogenic cell death (ICD) triggers an immune response. ICD is a novel concept that has emerged during the last decade. ICD depends on the concomitant generation of reactive oxygen species (ROS, Type I) and activation of endoplasmic reticulum stress (ER stress, Type II) (14, 15) to function as “enabler” and “eat me” signals to recruited immune cells (16–18). ICD has emerged as an important sign of a favorable immunogenic TME that provides the various functional immunological cell infiltration and cytokines (15, 19). Clinical studies have suggested that pre-treatment with ICD inducers sensitizes cells to immune checkpoint blockade treatment (20). Though discussed frequently, hyperthermia is a kind of ICD inducer. Below, we will discuss hyperthermia-induced ICD from two aspects including ICD-related biological events (ER stress, ROS, and apoptosis) and the accompanying generated damage-associated molecular patterns (DAMPs) with an emphasis on HSP.

### Hyperthermia-Induced ICD Depends on ER Stress and ROS

“Fever”-induced apoptotic, necrotic, or even live cancer cells constitute a relevant natural mode of tumor-associated antigen (TAA) (21, 22). Hyperthermia generates different modes of TAA depending on the temperature change. Generally, temperature at the fever range (37–41°C) leads to a protective function for cancer cells with presentation of their constituents, while temperatures of 41–43°C promote cell death predominantly by apoptosis with a balance between pro-apoptosis and anti-apoptosis. As temperature rises even higher, the pro-apoptosis becomes dominant. While temperatures rise above 43°C (thermal ablation range), tumor cells experience the destruction mainly by necrosis (23).

Thermal ablation induced necrosis is a pathologic cell death that can produce immunogenic inflammatory response (24). Unlike thermal ablation, fever range hyperthermia can only influence cell membrane fluidity and stability, change cell morphology, and influence intracellular sodium–calcium levels (25). At this temperature, the heat shock response and ER stress can occur simultaneously. Heat shock response-induced HSPs can either diminish the activation or relieve ER stress by activating a negative feedback system of the unfolded protein response (UPR) to avoid excessive activation (26) and can protect tumor cells against both caspase-dependent and caspase-independent apoptosis triggered by oxidative stress (27). Additionally, eIF2*α* phosphorylation, the hallmark of ICD (28, 29), was rarely induced at this temperature (30). While temperature rises between the “fever range” and “thermal ablation range” at 41–43°C, tumor cells died predominantly by apoptosis with a balance between pro-apoptosis and anti-apoptosis. This process involves the induction of CHOP, the alterations in calcium levels and the activation of ER proteases, calpain–calpastatin proteolytic system and caspase mediated apoptosis (30, 31). This process also accompanies with the upregulation of eIF2*α* phosphorylation. While both low (43°C) and high (45°C) hyperthermic exposures were capable of inducing cell death by activating apoptotic pathways, mild hyperthermia (43°C) triggers the apoptotic response in a more regulated manner in order to sustain apoptotic cell death (31).

Traditional view holds that apoptosis is non-immunogenic and does not induce an inflammatory response. However, recent studies have suggested that certain kinds of treatment that induce tumor cell apoptosis can also release DAMPs and induce ICD. Calreticulin (CRT) exposure, high mobility group box 1 (HMGB1) release, and adenosine triphosphate (ATP) secretion are essential factors for cell death to be considered ICD (32). In fact, heat-shock conditioning of cancer cells increased their CRT plasma membrane translocation and induced the release of HMGB1 protein. Moreover, both CRT and HMGB1 mobilization were associated with enhanced antigen cross-presentation and antigen present cell maturation after hyperthermia at mild temperature range of 41–43°C (33, 34). It remains to be elucidated that hyperthermia related apoptosis can induce ICD, but apoptosis induced by hyperthermia is involved in the ICD generation (35–37). Nevertheless, considering the ICD-related biological events of ER stress, ROS, and apoptosis and the accompanying generated DAMPs, hyperthermia can be regarded as an ICD inducer as well as other treatments (32, 38). Whereas, it should be noted that hyperthermia-induced ER stress or apoptosis is fostered by focused ROS rather than by secondary or collateral ER stress effects, which were thought to be more effective for ICD-associated immunogenicity (15). Moreover, this referred ICD is different from pathologic necrosis cell death caused by tumor ablation (39).

### HSPs Are Among the Most Important DAMPs Induced by Hyperthermia

Hyperthermia induces various kinds of DAMPs, including HMGB1, CRT, and ATP. In addition, study by proteomic profiling found quantitative proteins regulated by heat shock treatment that can be described as potential DAMPs or candidates for further immunological analysis (40). However, the current paradigm of the immunogenicity of hyperthermia mainly relies on HSPs and activated Toll-like receptor-4 (TLR-4) signaling pathways for the initiation of tumor-specific immune responses (38, 41). Here, we discuss various forms of HSPs and the suitable temperature for maximized immunity.

HSPs are a group of highly conserved chaperone proteins synthesized under pressure in a wide range of tumor cells containing HSP70, HSP60, HSP90, and small HSPs. Elevated HSPs are usually associated with poor prognosis in most cancer types. However, these overexpressed HSPs after hyperthermia are also associated with enhanced immune response. There are three forms of HSP: intracellular, membrane, and extracellular HSPs. Intracellular HSPs promote the maintenance of the innate structures and functions of their client proteins by facilitating protein folding when the cells are under homeostatic challenges (42). Unlike intracellular HSPs, studies found that small fractions of several heat-stress cognates are located at or near the cytoplasm inside the membrane along with cytoskeletal proteins, and that additional submembranous localization of HSPs may be a part of cellular responses to heat that associated with membrane damage (43). Whereas, later research found that this membrane HSP70 might also serve as a tumor-specific target for the cytolytic attack of CD56^bright^/CD94^+^ natural killer (NK) cells (25, 44). While, extracellular HSPs released from tumor cells are regarded as potent adjuvants to facilitate the presentation of tumor antigens and the induction of anti-tumor immunity (45, 46).

In accordance with relationship between biological events and temperature, HSPs start to release at 41°C and reach a maximum at 43°C but begin to diminish at 45°C (47). To achieve the optimal extracellular HSP synthesis for anti-tumor immune activation, Lin et al. developed a model to predict the optimal temperature and exposure time by involving factors such as different cell lines, cell incubation times, and heat administration methods into the model. They found that the maximum extracellular HSP synthesis was at 43°C, so was the maximum modulatory effect for tumor regression and decreased metastasis. When the temperature was further increased, HSP synthesis decreased, and the immune modulatory effect of hyperthermia was also downregulated (48). Whereas, through bioinformatic approach, Duzgun et al. identified a series of molecules that determine the thermoresistance and immunogenic cell death in thermotherapy through estimating the percentage of the two kinds of denatured proteins. They found that thermoresistance along with ICD both existed in a broad temperature windows, and that average *T*_m_ (50% of the protein is unfolded) of DAMPs (63.42°C) is remarkably higher compared to the thermal ablation temperature due to the function to interact with their pattern recognition receptors (PRRs) even under thermal stress (49). Although the suitable temperature for maximized immunity remains unclear, these models offer ways to rationally explore suitable conditions to exploit hyperthermia.

## Hyperthermia Enhanced the Immune Response in Multiple Steps

With increased tumor mutation burden and ICD, immunity is either activated or enhanced by hyperthermia for the subsequent immune response in multiple steps of the cancer-immunity cycle. Hyperthermia-activated immunity appears to be specific, persistent, and memorable. This activated immunity not only makes hyperthermia a capable treatment to defend against cancer but also orient it as a reliable treatment that can facilitate the efficacy of ICIs.

### Hyperthermia Promotes APCs’ Activation

It has been established for years that mild thermal stress regulates DCs’ activities to control infections and tumor growth (50, 51). Although APCs’ activation is not directly associated with the prognosis of PD-1/PD-L1 mAb treatment, APCs’ activation directly influences tumor-specific T cell responses. APCs’ activation includes antigen presentation and APC maturation. During the process of antigen presentation, hyperthermia mainly regulates “phagocytosis checkpoints” by enhancing the immunogenic “eat me” signals and repressing tolerogenic “eat me” signals as well as “do not eat me” signals (52). Specifically, phagocytosis of APCs is mainly enhanced by immunogenic “eat me” signals of DAMPs through receptor-mediated endocytosis *via* PRR. Moreover, hyperthermia represses the “do not eat me” signal through decreasing the expression of CD47 in the cell surface (53). In addition, hyperthermia can also inhibit tolerogenic “eat me” signals by transforming immature APCs and/or APCs exhibiting immunosuppressive phenotypes (M2 macrophages, N2 neutrophils, myeloid-derived suppressor cells) to a relatively mature one (54, 55). This transformations include infiltrating activated monocytes into the tumor microenvironment (56), inducing immature DCs to differentiate into DCs (45), promoting macrophage polarization to the M1 type that exerts pro-inflammatory effects, and promoting the release of inflammatory factors (57, 58). In fact, significantly increased phagocytosis rates of macrophages and DCs have been seen; moreover, this process seems to be temperature sensitive (>43°C).

Along with antigen presentation, APCs initiate a process of maturation by increasing the expression of MHC I, MHC II molecules and costimulatory molecules, and migrating to the draining lymph node. This process can be mediated by antigen presentation, TLR agonists, the standard maturation cocktail of pro-inflammatory cytokines (59), and physiological temperature stress of 40–41°C (50, 51). Traditional views hold that hyperthermia-induced ICD is among the strategies to improve the efficacy of dendritic cell-based immunotherapy for specific cancer types (60). However, studies have suggested that merely heating tumor cells cannot activate immature DCs. Only when tumor cells and immature DCs are both under sequential hyperthermia treatment, can the immature DCs be effectively activated. This result suggests that DCs’ maturation not only depends on danger signals with HSPs but also on hyperthermia itself independently (61). Further studies proposed that fever-range hyperthermia promote DCs from a quiescent status to an activated status by promoting the metabolic reprogramming in them (62, 63). The authors proposed that hyperthermia increased the expression of insulin-like growth factor binding protein 6 (IGFBP-6) and HSP70, whose autocrine mechanism increases the glycolysis, decreases the activity of the mitochondrial respiratory chain and consequent oxidative phosphorylation (OxPhos), enhances the production of NO and ROS, and promotes the mitochondrial Ca^2+^ overload. It should also be noted that this metabolic reprogramming of DCs functions more like a kind of checkpoint in DCs’ activation or maturation, and this process is an early event for the accomplishment of cell-specific immunologic adaptation.

### Hyperthermia Corrects Dysfunctional CD4 T Cell Immune Response

CD4 T cells display a large degree of plasticity to differentiate into Th1, Th2, Th17, and regulatory T cells (Tregs) in response to different tumor environments (64, 65). Th1 cells along with its generated chemokines exert prominent anti-tumor activity by blocking the formation of new blood vessels as well as promoting recruitment of tumor-killing immune cells. Intra-tumoral FoxP3 + Tregs impede effective immune response against cancer and impaired the efficacy of PD-1/PD-L1 mAb. In contrast to function of Tregs, Th17 cells may be prominent candidates for adoptive T-cell therapy (66–68). Functional systemic CD4 T cell immunity is essential for effector cytotoxic T lymphocyte (CTL) priming, memory CTL development, and effective PD-1/PD-L1 blockade (69, 70). Hyperthermia showed the potential of correcting dysfunctional CD4 T cell immune response by drifting CD4 T cells to Th1 and transforming Treg cells into Th17 cells to rebuild a favorable TME that can effectively respond to anti-PD-1/PD-L1 mAb.

DC maturation induced by thermal therapy is a prerequisite for CD4 T cell differentiation (57, 71). Besides fever-range hyperthermia (39–40°C) inhibits Th2 and Treg growth, induces spleen Th1 and Tc1 proliferation, and promotes Th1 cell-associated secretion of IL-2, IFN-*γ* and TNF-α in spleen (72). While cryothermal therapy not only reduced the percentage of Tregs and myeloid-derived suppressor cells (MDSCs) in spleen, lung and blood but also promoted CD4^+^ T cell’s differentiation into predominant CD4^+^ CTL, Th1, Th2, and Tfh subsets (73). Moreover, compared with radiotherapy alone, combined radiotherapy with hyperthermia regulated the tumor microenvironment and upregulated the Th1/Th2 ratio (74). In addition, HS-TEX can elicit Th1-polarized immune responses by increasing the production of IgG2a and IFN-*γ* in sensitized tumors (75). Besides, preclinical studies have shown the potential of hyperthermia to promote Th1-related immunity and repress the function of Treg cells. Last, the correcting of dysfunctional CD4 T cell by hyperthermia has also been proven by that patients with tumor treated with hyperthermia showed increases in Th17 cells and decreases in Tregs in the peripheral blood (76).

### Hyperthermia Creates a Favorable Inflammatory Tumor Microenvironment

It is highly dependent on cytokines and chemokines for “cold” tumors with low response rate to PD-1/PD-L1 mAb to transform to a “hot” one that is infiltrated with immune cells in tumor sites. Serum cytokine analysis revealed that hyperthermia at 41°C for 30 min induces an intratumoral inflammatory cytokines and chemokines to increase in enhanced T-cell trafficking (77). Specifically, mild hyperthermia increases the expression of L-selectin, P-selectin, and intercellular cell adhesion molecule-1(ICAM-1) in the vessel wall (78–80) and drives the production of a number of pro-inflammatory cytokines and chemokines (*i.e.* interleukin−1β, IL-6, IL-8, IL-10, and CCL22) (77, 81). This inflammatory cytokines and chemokines act at multiple discrete steps that favor lymphocyte infiltrate to the tumor microenvironment and attack solid tumors in the immune cascade.

Among cytokines and chemokines induced by thermal stress, IL-6 plays a pivotal role in the tumor immune microenvironment. Specifically, cryo-thermal therapy-induced IL-6-rich acute pro-inflammatory response promotes DC phenotypic maturation, CD4(+) T cell differentiation, and Th1 anti-tumor immunity (71, 82). In addition, hyperthermia induces M1 macrophages to secrete CXCL10 and IL-6 to induce CD4 T cell differentiation into Th1 and CD4 CTL cells, and reduce MDSC aggregation (57). Moreover, IL-6 stimulated by HS-TEX promotes Treg transformation to Th17 cells and induces CD4 T and CD8 T cell-dependent immune responses (76). Though IL-6 also drives tumor growth and promotes survival of neoplastic cells, these tumor-promoting activities are completely counteracted by the effect of T lymphocyte infiltration into the tumor site with a result of tumor cell killing and tumor regression (81, 83). However, most of the research was conducted under physiological temperature stress of 40–41°C. Moreover, hyperthermia alone seems insufficient for tumor cell regression for the result that combination of an artificial cytokine storm and hyperthermia rather than hyperthermia itself can effectively promote the anti-tumor response (84).

### HS-TEX Extracted After Hyperthermia Acts as Tumor Vaccine

Exosomes are small membrane vesicles of endocytic origin that have a typical bilayer-membrane structure shuttling from donor cells to recipient cells to communicate and transport information between different cells. In response to a variety of stress conditions, cells employ extracellular vesicle to transmit a pro-survival message in the tumor microenvironment for evasion of cell death and transmitting resistance to therapy (85). Heat stress not only promotes the release of TEX (quantity) (86) but also promotes TEX to pack with more positive immunomodulators (HSP70, adhesion molecules, chemokines) rather than negative regulators (fasL, TGF-*β*) (87).

**Figure 1:**
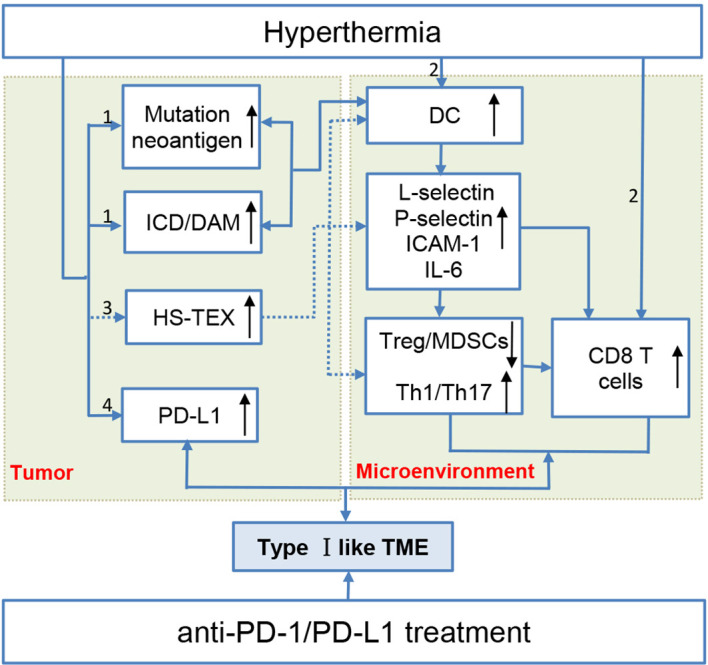
Hyperthermia creates a type I-like tumor microenvironment, and the multifaceted mechanisms make hyperthermia a potent immune checkpoint inhibitor sensitizer. (1) Hyperthermia increases the tumor mutation burden/neoantigen and promotes immunogenic cell death. These two aspects promote DC activation maturation and thus transform the immunosuppressive microenvironment by inhibiting Treg cells and promoting tumor-infiltrating lymphocyte recruitment. (2) Hyperthermia can directly promote DC and T cell maturation. (3) Exosomes extracted from heat-stressed tumor cells (HS-TEX) act as a cancer vaccine to activate DCs and promote cells to secrete IL-6 to transform the immunosuppressive TME (marked with dotted lines) (4) Hyperthermia can upregulate PD-L1 expression in an elevated temperature.

HS-TEX is a reliable tumor vaccine for tumor-specific immune response. A study suggested that HS-TEX extracted from ascites of gastric cancer can induce DCs’ differentiation and promote tumor-specific immune response (88). Whereas, intra-tumoral injection of HS-TEX derived from colon cancer cells and B lymphoma with hyperthermia efficiently induced tumor-specific anti-tumor immunity in mouse models (89, 90). Besides, HS-TEX can activate DCs to release IL-6 to trigger subsequent transformation of the immune microenvironment to reduce Tregs and promote the chemotaxis of T cells to tumors (76). Contents in the HS-TEX plays an important role for its function in TME. HSP-70 abundant exosomes recruit more NK cells and promote the killing of NK cells better than that of apoptotic fragments and HSP-70 knockout exosomes (91). Chemokines in HS-TEX recruit and activate DCs and tumor-specific T cells through a lipid raft-dependent pathway to promote tumor immune response. Despite the promising role of HS-TEX in TME, a study also suggested a bystander effect induced by HS-TEX from tumor cells that promotes the survival of unstressed ones (12). Moreover, PD-L1 can also express in TEX for immune evasion, but this expression cannot be neutralized by anti-PD-L1 mAb treatment (92). The sophisticated role of HS-TEX in the TME remains to be elucidated *in vivo* rather than as a vaccine. (Thus, this part is presented in **Figure 1** marked with dotted lines.)

### Hyperthermia Promotes CD8 T Cell’s Quantity and Quality

Despite the presence and activation of several immunologic components in the TME, tumor cells are not easily eradicated (93). Mechanisms involved in this impaired response are attributed to the immune suppressive agents in the TME, including the depletion of naïve anti-tumor T cells during thymic lymphocyte development, unresponsiveness of CTLs due to impaired of costimulatory or enhanced coinhibitory molecules, prolonged presence of immunosuppressive cells and along with secreted inhibitory molecules from those cells (94). Consequently, although immune cells are found in the TME, they are not fully effective (95, 96). Hyperthermia can break this barrier by promoting antigen-specific naive CD8 T cell differentiation, enhancing the cytotoxic potential of T cells and promoting memory stem T cell generation.

CD8 T cells’ differentiation and function of cytotoxicity are both temperature-sensitive events. Study researched the number of Melan-A/Mart-1-specific CD8 T cells in patients after isolated limb perfusion with hyperthermia and found a small increase in tumor-specific T-cells in a subpopulation of patients with melanoma, demonstrating the potential of thermal therapy in the activation and differentiation of immune effector cells in the tumor microenvironment (97). Another study showed that heat-shocked pre-treated melanoma cell lysates promote the proportion of a prototypic effector T cells (PD-1^lo^CD8 T cell) in the TME to prevent dysfunctional T-cell accumulation and inhibit tumor growth (98). The result that hyperthermia promotes naive CD8 T cells’ differentiation are also proved by a study that CD8+ T cells under heat stress (39.5°C) can differentiate into effector cells by reversible clustering of GM1(+) CD-microdomains in the plasma membrane, clustering of TCR*β* and the CD8 coreceptor, and enhancing the rate of CD8+ T cell-APC conjugate formation in all spleen, lymph nodes, and peripheral blood T cells. While during the phase of cytotoxicity, the ability IFN-gamma production and cytotoxicity effect of effector CD8+ T cell are also enhanced after hyperthermia (99, 100). The enhanced cytotoxicity effect is associated with the expression of HSF1 that upregulates fas ligand expression by translocation of the transcription factors AP-1 and NF-*κ*B (101). Some researchers believe that exhaustion of CTLs is due to impaired formation of memory T cells (102). Studies have shown that hyperthermia induces differentiation of CD8^+^ T cells into memory stem T cells (T_SCM_) (103) and could possibly redistribute the memory T cells of patients with tumor (104). Thus based on the results of enhanced T-cell trafficking and promoted CD8 T cell immunity, hyperthermia can effectively target at the TME to edit the immunity for cancer treatment.

## Hyperthermia Promotes the Expression of Coinhibitory Molecules

Despite the reliable effects of hyperthermia on the immunogenicity and immunoreactivity of tumors, hyperthermia can also upregulate the expression of coinhibitory molecules. Studies have shown that heating in the range of 37–49°C successively upregulated the expression of PD-L1 and IDO on the surface of tumor cells. They found that with time, the expression of IDO increased at 48 h after the heat treatment and then decreased at 72h. Whereas, the expression of PD-L1 have the highest expression at 72 h. Moreover, the upregulated PD-L1 expression not only showed in tumor margins but also in distant tumors after hyperthermia (ablation temperature) (105–107). It remains to be elucidated that the upregulated coinhibitory molecules of PD-L1, PD-1, and Tim-3 are a cellular protective responses to avoid excessive immune activation or a byproducts from heat shock response when cells under damage. It should be noted that the upregulated coinhibitory molecules on tumor cells can also lead to impaired function of CD8 T cell. However, this upregulated immune checkpoint molecule can be neutralized by ICIs, and synergetic effect has been achieved for the combined hyperthermia and ICIs for tumor remission (see the following section). Thus, based on the foregoing reference that hyperthermia can either activate or enhance the immune response and upregulate PD-L1 in several kinds of tumors, we propose that treatment with hyperthermia creates a type I like tumor immune microenvironment with tumor infiltrating lymphocytes (TILs) and upregulates PD-L1 to work in complement with ICIs for cancer treatment (**Figure 1**).

## Preclinical Data Combining Hyperthermia and ICIs

Discussed and researched for centuries, hyperthermia is seldom applied as a mainstream therapy or an adjuvant approach for cancer therapy. Clinical studies have researched the combination treatment of hyperthermia with cytokines and DCs; however, the results are conflicting. The reasons are mainly attributed to tumor tissue selection, antigen load *in vitro* and *in vivo*, and whether DCs could be recruited effectively. Despite the conflicting results of hyperthermia with traditional immune treatment and the constraint of antigen masking or shielding, thermoresistance, the bystander effect of HS-TEX and possible high expression of PD-L1 in HS-TEX, preclinical research of the combination regimens involving hyperthermia and ICIs has achieved optimistic results. However, hyperthermia is mainly restricted to nanoparticle-mediated hyperthermia and radiofrequency ablation.

Nanoparticle-mediated hyperthermia is a localized non-invasive treatment with controllable irradiation that has emerged as a new paradigm towards precise cancer therapy. Nanoparticle-mediated hyperthermia includes photothermal therapy and magnetic hyperthermia. Studies have found that the combination of ICIs (CTLA-4, PD-L1, IDO) and nanoparticle-mediated hyperthermia can promote antigen capture, enhance ICD effect, inhibit Treg cells’ function, promote M1 macrophages’ differentiation, recruit several folds of tumor-infiltrating lymphocytes, and achieve lasting memory for the inhibition of tumor growth in primary and distant sites (106–114). The synergetic role of heat and ICIs is further confirmed by results that enhanced tumor antigen-specific T cell responses and an increased Teff to Treg ratio in distant tumors with a combination of RFA and anti-PD-1 mAb administration (105). Despite the favorable results, several studies have shown limited results for complete tumor remission for ICIs with hyperthermia. Thus, triple combination strategies including ICIs with radio-/chemotherapy or TLR agonists have been studied, and they have also achieved favorable results with decreased tumor volume, increased metastatic dissemination, prevention of tumor rechallenge, and improved overall survival (115–126). A case report also found that hyperthermia and ipilimumab combined with IL-2 achieved complete clinical remission of stage IV triple-negative breast cancer with lung metastasis (127). Despite the favorable results, a preclinical study combining magnetic iron oxide nanoparticle hyperthermia and anti-PD-1 and anti-CTLA-4 with a 4T1-luc cell mouse model also showed decreased tumor volume but increased metastatic dissemination and no improvement in overall survival (128). In fact, a study has shown that tumors quickly overcame immune responses by inhibiting the function of CD8 and CD4 T cells, driving a shift to a higher Treg/Teff ratio and upregulating PD-L1/PD-1 expression, which result suggested that tumor microenvironment after hyperthermia is variable and is favorable for anti-PD-1/PD-L1mAb treatment for only a narrow time window (105). Thus, compared to similar studies mentioned above, the reasons may partially be attributed to the unsynchronized treatment of hyperthermia and ICIs, for which other reasons should be explored to avoid further clinical failure. Detailed information on the combination therapies is shown in **Table 1**.

**Table 1:** Preclinical studies involving hyperthermia and immune checkpoint inhibitors.

Hyperthermia	Immune checkpoint inhibitors	Temperature	Tumor (mouse model)	Reference
CuS NPs-PEG-Mal-mediated PTT	Anti-PD-L1 mAb	55°C	4T1 breast tumor	(108)
Mild photothermal	Anti-PD-L1 mAb	45°C	4T1 breast tumor and B16-F10 melanoma tumor	(107)
CoFe2O4@MnFe2O4 nanoparticle-mediated magnetic hyperthermia	Anti-PD-L1 mAb	50°C	4T1 breast tumor	(109)
FVIOs-mediated magnetic hyperthermia	Anti-PD-L1 mAb	43–44°C	Orthotopic 4T1 breast cancer	(110)
Au nanoparticle-loaded membrane nanosheet photothermal therapy	Anti-PD-L1 mAb	64.4 ± 1.4°C	B16−F10 melanoma-tumor	(111)
GNPs-hPD-L1 siRNA-mediated photothermal therapy	Nanoprism-assisted PD-L1 siRNA	41.2°C	HCC827 lung cancer cell bearing tumor	(112)
APP- and HAuNS-loaded PLGA nanoparticle photothermal ablation	Sustained release anti-PD-1 peptide	50–55°C	4T1 breast tumor and CT26 tumor	(113)
Au@Pt-LMDP conjugated photothermal-immunotherapy	Release of a D-peptide antagonist of PD-L1	+ 20°C	4T1 breast tumor	(114)
NLG919/IR780 micelle-mediated PTT	IDO inhibitor	54°C	MCF-7 breast cancer	(106)
**Triple combination**				
mPEG-Pep-IDOi/ICG NPs-mediated phototherapy	Anti-PD-L1 mAb and nanoplatform of IDO inhibitor (IDOi)	Maximum 60°C	B16–F10 melanoma tumor	(115)
Magnetic iron oxide nanoparticle hyperthermia	Anti-PD-1 mAb and anti-CTLA-4 mAb	43°C	4T1-luc breast cancer model	(128)
PEG−rGO−FA−IDOi-mediated PTT	Anti-PD-L1 mAb and IDOi	53°C	CT26 colorectal cancer	(116)
PDMN-JQ1 nanoplatform-mediated photothermal therapy	bromodomain and extra-terminal inhibitor JQ1 downregulated the expression of PD-L1 and inhibited the BRD4-c-MYC axis	+21.7–20.3°C	4T1 breast tumor	(117)
PDA-PEG-R848-CD nanoparticle PTT	Anti-PD-L1 mAb + PDA loaded with TLR7 agonist	52.4°C	4T1 breast tumor	(118)
Fe3O4-R837 SP-involved PTT	Anti-PD-L1 mAb and nanoparticles loaded with Toll-like receptor 7 agonist	Ablation temperature	4T1 breast tumor	(119)
Iron nanoparticle-mediated magnetic hyperthermia	Anti-CTLA-4 mAb and TLR7 agonist	55°C	CT26 mouse colon cancer and murine B16 skin melanoma	(120)
WO2.9-WSe2 nanoparticles l RT/PTT	Anti-PD-L1 mAb-based CBT + low radiation dose	48°C	4T1 breast tumor	(121)
COF@ICG@OVA PTT/PDT therapy	Anti-PD-L1 mAb + PDT + PTT	>63.5°C	H22 murine hepatoma	(122)
Hyaluronic acid-shelled PPy/CPT nanoparticles	Anti-PD-L1 mAb and camptothecin	45–50°C	4T1 Breast cancer	(123)
FA-CD@PP-CpG phototherapy	Anti-PD-L1 mAb+low dosage of loaded docetaxel	44°C	4T1 breast tumor	(126)
Pd-Dox@TGMs NPs chemical-photothermal therapy	Anti-PD-L1 mAb and doxorubicin	51.2°C	CT26 colorectal cancer	(124)
Cu-PPT + 650 + 808 nm laser photo/chemodynamic therapy	Glutathione peroxidase-mimicking and PD-L1 mAb	–	CT26 colorectal cancer	(125)
Regional hyperthermia followed by systemic fever-range hyperthermia induced by interleukin-2	Ipilimumab (case report)	<42°C	Stage IV triple-negative breast cancer with lung metastasis	(127)
**Radiofrequency ablation**				
Radiofrequency ablation	Anti-PD-1 mAbs	>45°C	CT26 mouse colon cancer	(105)
	Atezolizumab (case report)	>45°C	Stage IV non-small cell lung cancer	(129)

## Conclusions and Future Challenges

Providing danger signals and reforming immune cells in the TME, hyperthermia is involved in multiple steps of regulating pathways in the cancer-immunity cycle; the immunomodulatory effect not only makes hyperthermia a treatment capable of defending against cancer but also makes the regimens of hyperthermia and ICIs a promising treatment for clinical use. Two of the major concerns are whether this combination is sufficient for the initiation and clearance of the tumor and that the combination would not drag the result in the opposite direction, *i.e.*, the super-progression of the tumor due to immunotolerance. For the first concern, selective combination with the known treatment would be a way for solution. In fact, both radiotherapy and chemotherapy can directly kill tumor cells, whose cell debris can be recognized as a tumor *in situ* vaccine that can promote the effect of ant-PD-1/PD-L1 mAb. Moreover, hyperthermia is a potent radio-/chemo-sensitizer *via* a series of supplementary cytotoxic effect (130). Thus, it is expected for efficacy of the triple combination of anti-PD-1/PD-L1 mAb, hyperthermia and radio/chemotherapy in clinics. Moreover, it also seems promising for PARPi, anti-angiogenesis treatments and other treatments to substitute radio/chemotherapy to combine with PD-1/PD-L1 mAb and hyperthermia for the treatment of tumors with low mutation, fewer neoantigens or disorganized tumor vessels. Thus, rational different combination therapies are promising for the eradication of tumors (131). For the second question, the authors believe that the patients should also be explored and selected for the combination, which point is also important for the first concern. Studies have found that certain gene mutations, such as *KRAS*, are more sensitive to hyperthermia as they exhibit sustained ERK signaling hyperactivation and increased Wingless/Integrated (WNT)/beta-catenin signalling (132). Moreover, using a bioinformatic approach, a series of molecules have been identified as determinants of resistance/sensitivity to thermotherapy (49). The results of the two studies offer ways for accurate selection when treated with hyperthermia. Last but not least, thermoequipment and procedures should be normalized with schedules based on the model system, the magnitude, the duration of the thermal stress, and the time of recovery after heat exposure (133). However, radiofrequency and local hyperthermia are the most commonly used hyperthermia regimens in clinical practice; they exploit ablation and mild temperature for treatment directly instead of the help of particle media. How they can be properly used with ICIs and whether they can achieve equally promising results remain to be elucidated.

## Author Contributions

ZL performed the manuscript preparation and drafted the manuscript. JD helped draft the manuscript. JS revised the manuscript and approved the final version. YM contributed to the conception and design of the current study, revised the manuscript, and approved the final version. All authors contributed to the article and approved the submitted version.

## Conflict of Interest

The authors declare that the research was conducted in the absence of any commercial or financial relationships that could be construed as a potential conflict of interest.
